# Impact of tumor and node stages on the efficacy of adjuvant oxaliplatin-based chemotherapy in stage III colon cancer patients: an ACCENT pooled analysis☆

**DOI:** 10.1016/j.esmoop.2025.104481

**Published:** 2025-03-04

**Authors:** R. Cohen, M. Raeisi, B. Chibaudel, G. Yothers, R.M. Goldberg, J.-B. Bachet, N. Wolmark, T. Yoshino, H.-J. Schmoll, D.G. Haller, R. Kerr, S. Lonardi, T.J. George, E. Shacham-Shmueli, Q. Shi, T. André, A. de Gramont

**Affiliations:** 1 Sorbonne University, Department of Medical Oncology, Hôpital Saint-Antoine, AP-HP, and INSERM UMRS 938, Équipe Instabilité des Microsatellites et Cancer, Équipe Labellisée par la Ligue Nationale Contre le Cancer, SIRIC CURAMUS, Centre de recherche Saint Antoine, Paris, France; 2 Statistical Unit, ARCAD Foundation, Paris, France; 3 Department of Medical Oncology, Franco-British Hospital, Levallois-Perret, France; 4 Department of Biostatistics, University of Pittsburgh and NRG Oncology, Pittsburgh, USA; 5 Department of Oncology, West Virginia University Cancer Institute, Morgantown, USA; 6 Hepato-gastroenterology and Digestive Oncology Department, Sorbonne University, Pitié Salpêtrière Hospital, APHP, Paris, France; 7 NSABP Foundation, Pittsburgh, USA; 8 Department of Gastroenterology and Gastrointestinal Oncology, National Cancer Center Hospital East, Kashiwa, Japan; 9 Division of Clinical Research in Oncology, Martin-Luther-University Halle-Wittenberg, Halle (Saale), Germany; 10 Abramson Cancer Center, University of Pennsylvania, Philadelphia, USA; 11 Department of Oncology, University of Oxford, Oxford, UK; 12 Medical Oncology, Veneto Institute of Oncology IOV-IRCCS, Padua, Italy; 13 Division of Hematology and Oncology, University of Florida, Gainesville, USA; 14 Sheba Medical Center at Tel-Hashomer, Tel Aviv University, Tel Aviv, Israel; 15 Department of Quantitative Health Science, Mayo Clinic, Rochester, USA; 16 ARCAD Foundation, Paris, France

**Keywords:** colon cancer, stage III, chemotherapy, oxaliplatin

## Abstract

**Background:**

Standard adjuvant treatment of stage III colon cancer (CC) is fluoropyrimidine with oxaliplatin. Recently, stage III was subdivided into low-risk (T1-3, N1) and high-risk (T4 and/or N2), with the benefit of adding oxaliplatin varying across these substages. In this study, we aimed to assess the impact of oxaliplatin on survival outcomes in subdividing stage III CC patients based on T and N staging.

**Patients and methods:**

A total of 4942 stage III CC patients were pooled from the three randomized pivotal trials of oxaliplatin. Kaplan–Meier curves, Cox models stratified by study, and interaction tests were used to assess the oxaliplatin effect across subgroups based on T and N stages. The primary endpoint was overall survival (OS).

**Results:**

The prevalence of tumor stages was T1-2 12.4%, T3 74.4%, and T4 13.1%; nodal stages were N1 64.7% and N2 35.3%. A significant OS benefit from oxaliplatin was seen only in T3 (5-year OS = 77.2% versus 73.0%, *P* < 0.001): T3N1 (hazard ratio 0.72, 95% confidence interval 0.62-0.85, *P* < 0.001) and T3N2 (hazard ratio 0.81, 95% confidence interval 0.69-0.95, *P* = 0.010). No benefit was observed for T1-2 (5-year OS = 87.8% versus 88.7%, *P* = 0.644) or T4 patients (5-year OS = 62.6% versus 60.2%, *P* = 0.648). Subgroup analysis revealed a significant interaction between T stage and the effect of oxaliplatin treatment on OS, whereas no such interaction was observed for N stage.

**Conclusions:**

Our analysis revealed that oxaliplatin-based chemotherapy offers a significant survival benefit in stage III CC patients with T3 tumors. In contrast, no survival benefit was observed for T1-2 or T4 patients. These results suggested that T stage plays a more crucial role than N stage in predicting treatment benefit, highlighting the need for tailored treatment strategies based on tumor characteristics.

## Highlights

• T stage is a more critical factor than N stage in evaluating oxaliplatin-based adjuvant therapy for stage III colon cancer.
• Oxaliplatin-based adjuvant therapy is recommended specifically for T3 patients.
• These findings underscore the importance of personalized treatment strategies based on tumor characteristics.

## Introduction

Since 2004, the standard adjuvant treatment after surgery for colon cancer (CC) with lymph node involvement (stage III) has been chemotherapy combining a fluoropyrimidine (FP) and oxaliplatin (OX), with one of two regimens: FOLFOX or CAPOX. This standard adjuvant treatment was established by three pivotal studies: MOSAIC, NSABP C-07, and XELOXA.^1^, ^2^, ^3^ The MOSAIC and C-07 studies included patients with both stage II (without lymph node invasion) and stage III CC, while the XELOXA included exclusively stage III patients.

For stage III patients, the absolute increments observed as a consequence of adding OX to FPs in 5-year disease-free survival (DFS) were 7.3%, 6.6%, and 6.3% in MOSAIC, C-07, and XELOXA, respectively, while the 5-year benefits in overall survival (OS) were 4.3%, 2.7%, and 3.4%, respectively.^3^, ^4^, ^5^ This suggests that ∼15 patients need to be treated with OX to have one additional patient free of disease at 5 years. These studies, individually and in a 2016 ACCENT meta-analysis^6^ could not identify a subgroup that did not benefit from OX, although some caveats were suggested for elderly patients and those with small tumors (T1-2). This ACCENT meta-analysis evaluating the OS benefit of OX was carried out on the overall population, however, including stage II and III patients in the MOSAIC and C-07 studies.^6^ This approach might have masked the outcomes in stage III patients, as it has been shown that OS is not improved by the addition of OX in the adjuvant treatment of stage II patients.^7^

Recently, the IDEA study investigated reducing the duration of chemotherapy with FP and OX for stage III CC.^8^ In this study, stage III CC was pragmatically divided into low risk (T1 to 3, N1 with fewer than four lymph nodes invaded) and high risk (T4 and/or N2). The low-risk group had a 3-year DFS of ∼80%, while the high-risk group with T4 or N2 tumors, had a 3-year DFS of ∼60%. Non-inferiority of 3 months of therapy compared with 6 months was not confirmed in the overall population. For patients treated with CAPOX, however, 3 months of therapy was as effective as 6 months, particularly in the lower-risk subgroup, in terms of DFS and OS.^8^^,^^9^ Following the publication of the IDEA study, two studies were conducted to examine the impact of OX in substage III groups as defined by the IDEA study.^10^^,^^11^ The benefit of OX in both low-risk and high-risk cancers was confirmed. A difference in benefit was observed within the two high-risk subgroups (T4 and N2), however, with a benefit noted in the N2 subgroup and little or no benefit noted in the T4 subgroup.^11^

This prompted us to carry out this study to assess the relative impact of OX by subdividing stage III patients based on T and N staging and to investigate the factors within each subgroup that might explain differences in OX impact.

## Methods

### ACCENT database

The ACCENT database contains patient-level information on >55 000 patients enrolled on 34 adjuvant trials since 1977. All clinical trials included had institutional review board approval. Endpoints in the studies included in the ACCENT database include OS, DFS, and recurrence-free survival. The present analysis received the approval of the ACCENT Scientific Committee.

### Study population

Data from the patients enrolled in three studies from the ACCENT database that compared adjuvant OX-based chemotherapy with treatment with an FP alone were pooled. MOSAIC and C-07 included 4654 stage II and III CC patients who received either an FP alone regimen including 5-fluorouracil plus leucovorin or the same FP regimen plus OX (FPOX).^1^^,^^2^ Patient inclusion criteria were the same in both studies except for age (limited to 75 years in MOSAIC) and the definition of the inferior extent of the tumor (tumors must have been at least 15 cm from the anal margin in MOSAIC and at least 12 cm in C-07). In both studies, the fluorouracil dose-intensity was the same between the investigational and the control arm. In the XELOXA study,^3^ that included 1883 only stage III patients, a different FP regimen was used in the investigational OX arm, i.e. oral capecitabine named the CAPOX regimen, versus those used in the control arm, i.e. either weekly bolus fluorouracil plus leucovorin for 32 weeks^12^ or monthly 5 consecutive days fluorouracil bolus plus leucovorin for 24 weeks.^13^ To our knowledge, there are no direct comparisons of leucovorin and 5-fluorouracil (LV5FU) and capecitabine. The results of the two experimental arms of the AVANT trial (NCT00112918), FOLFOX and CAPOX in combination with bevacizumab, however, were similar. Based on these data, it is very unlikely that differences between the two FP regimens might impact our results. The clinical variables studied to determine their prognostic value were T stage, N stage, gender, primary tumor side, Eastern Cooperative Oncology Group (ECOG) performance status, degree of tumor histologic differentiation, body mass index (BMI), age, the presence or absence of perforation/obstruction, and the number of examined lymph nodes.

In this study, our focus was on stage III (T any, N1-2, M0) CC patients, analyzing the most recent survival data from the three studies.^4^^,^^5^^,^^14^ Additionally, stage II CC patients from the MOSAIC and C-07 studies were included for interaction analysis.

### Outcome variables

The main outcome variable used in all three studies was OS which was defined as the time from randomization to death from any cause. The secondary outcome variables were DFS defined as time to recurrence of the first CC (second primary colorectal cancer is discarded) or deaths of any cause and time to relapse (TTR) defined as the time from randomization to relapse or death from CC, whichever occurred first (second primary colorectal cancer, deaths without evidence of recurrence, deaths related to protocol treatment, deaths to non-CC were discarded).^15^ Survival after relapse (SAR) was also studied.

### Statistical analyses

This study was an exploratory pooled analysis with no formal hypothesis testing or power assumptions; all analyses and results are considered to be hypothesis generating rather than definitive. The population study was analyzed in the subgroups defined by T and N substages. Baseline characteristics were compared between treatment arms within each of these subgroups using the chi-square test or Fisher’s exact test, as all variables were categorized.

The univariable and multivariable analyses were then carried out to determine the effect of the individual prognostic factors within each of these subgroups. For the multivariate analysis, we faced the problem of missing prognostic factors data in the XELOXA study since only the MOSAIC and C-07 studies had comprehensive data on primary tumor location, the presence of perforation/obstruction, and the exact number of positive lymph nodes. For this reason, we studied two multivariate models including all variables selected by backward regression with a *P* value <0.1 in the univariable model, using the stratified Cox model, needed with the heterogeneity across trials: MODEL 1 with a smaller set of adjusting variables, but larger number of patients with the three trials, and MODEL 2 with a larger set of adjusting variables, but smaller number of patients with only two studies. The results from both models were then compared by assessing consistency in hazard ratios (HRs), confidence intervals (CIs), and statistical significance of the primary variables. Similar results between those two models, would reinforce the conclusions. Since the Cox model was stratified by arms within trials, however, the model using more variables might be more reliable because the model with more patients could not run properly with the missing variables as all patients in XELOXA would have had the same values for these variables.

To evaluate the benefit of the addition of OX to FP, survival was compared between patients receiving the OX-based regimen or those who did not. To assess this effect across patient subgroups based on T and N stages, the survival outcome distribution was estimated using Kaplan–Meier curves and Cox models stratified by study and interaction tests that were carried out between treatment and T and N substages among enrolled stage III patients. Furthermore, we carried out interaction tests between patient subgroups based on T stage from stage II and III pooled population and the effect of OX to confirm our results on the impact of T stage on OX benefit in stage III CC. A likelihood ratio test was conducted to compare nested models, one including T stage and another including N stage, to evaluate the relative contribution of each variable to model fit.

An interaction was considered as significant with a *P*_*int*_ <0.1, value chosen to gain power and not miss true interaction effects.^16^ All CIs were set at 95%. Statistical significance was assessed using a two-sided *P* value of <0.05, and HRs were considered clinically meaningful if they exceeded 1.15 or were <0.85, given the large cohort size. All analyses were carried out using R software (version 3.5.2).

## Results

The pooled population included 4942 stage III patients, 3677 (74.4%) T3 [2310 (46.7%) T3N1 and 1364 (26.7%) T3N2], 613 (12.4%) T1 and T2 (T1-2N1 10.1% and T1-2N2 2.3%) and 646 (13.1%) T4 (T4N1 7.8% and T4N2 4.3%), 3198 (64.7%) N1 and 1740 (35.3%) N2 patients. There were six missing patients for the T stage and four for the N stage (Table 1). The stage distribution was the same in both the FP and FPOX arms (Supplementary Figure S1, available at https://doi.org/10.1016/j.esmoop.2025.104481).

**Table 1:** Patient’s characteristics according to treatment in stage III patients from the pooled MOSAIC, C-07, and XELOXA studies.

	Without oxaliplatin (*n* = 2476) no. (%)	With oxaliplatin (*n* = 2466) no. (%)	Total (*n* = 4942) no. (%)	*P* value
Age, years				0.26
<70	2014 (81.34)	2037 (82.60)	4051 (81.97)	
≥70	462 (18.66)	429 (17.40)	891 (18.03)	
Gender				0.42
Women	1140 (46.04)	1106 (44.85)	2246 (45.45)	
Men	1336 (53.96)	1360 (55.15)	2696 (54.55)	
ECOG PS				0.52
0	1636 (66.21)	1605 (65.30)	3241 (65.75)	
≥1	835 (33.79)	853 (34.70)	1688 (34.25)	
Missing	5	8	13	
BMI				0.43
<30	1986 (80.40)	2004 (81.33)	3990 (80.87)	
≥30	484 (19.60)	460 (18.67)	944 (19.13)	
Missing	6	2	8	
Tumor sidedness				0.007
Left	895 (61.85)	820 (56.83)	1715 (59.34)	
Right	552 (38.15)	623 (43.17)	1175 (40.66)	
Missing	1029	1023	2052	
Differentiation				0.37
Poorly	438 (18.36)	415 (17.31)	853 (17.83)	
Well or moderately	1948 (81.64)	1982 (82.69)	3930 (82.17)	
Missing	90	69	159	
T stage				0.59
T1-2	318 (12.87)	295 (11.97)	613 (12.42)	
T3	1827 (73.94)	1850 (75.05)	3677 (74.49)	
T4	326 (13.19)	320 (12.98)	646 (13.09)	
Missing	5	1	6	
N stage				0.94
N1	1604 (64.83)	1594 (64.69)	3198 (64.76)	
N2	870 (35.17)	870 (35.31)	1740 (35.24)	
Missing	2	2	4	
Perforation/obstruction				0.90
No	1210 (78.93)	1202 (79.18)	2412 (79.06)	
Yes	323 (21.07)	316 (20.82)	639 (20.94)	
Missing	943	948	1891	
No. of examined lymph nodes				0.11
>12	793 (51.93)	831 (54.89)	1624 (53.40)	
≤12	734 (48.07)	683 (45.11)	1417 (46.60)	
Missing	949	952	1901	

ECOG PS, Eastern Cooperative Oncology Group (ECOG) performance status.

### Patients characteristics and prognostic factor analyses

#### All stage III population

**Figure 1:**
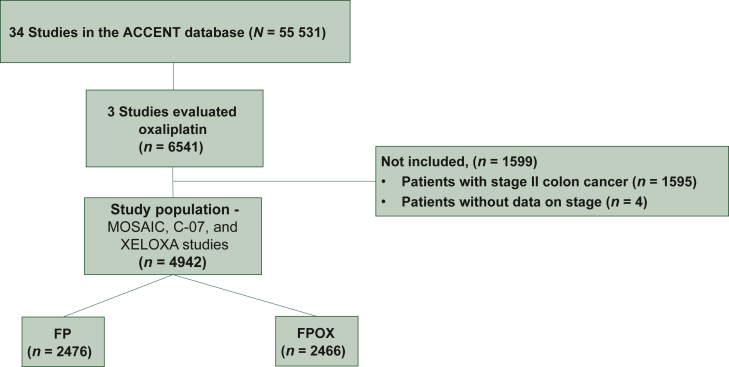
**CONSORT diagram.** FP, fluoropyrimidine; FPOX, fluoropyrimidine and oxaliplatin.

Detailed information regarding the population study used for evaluation of OX efficacy in stage III CC is provided in Figure 1 and Supplementary Table S1, available at https://doi.org/10.1016/j.esmoop.2025.104481. Patient characteristics were well-balanced according to treatment allocation, except in the frequency of right-sided tumors that were significantly more prevalent in the OX arm compared with the non-OX arm (43.17% versus 38.15%, *P* = 0.007) (Table 1).

For OS in the stage III population, the independent prognostic factors identified in the univariate analysis included T and N stages, primary tumor side, age, gender, differentiation, number of examined lymph nodes, perforation/obstruction, and treatment (Supplementary Table S2, available at https://doi.org/10.1016/j.esmoop.2025.104481). Among these, age, gender, and treatment (primary variables) were available across the three trials, forming the basis for MODEL 1. MODEL 2 included all independent factors, excluding the XELOXA study. Consistency of the primary variables across the two models was observed (Supplementary Figure S2, available at https://doi.org/10.1016/j.esmoop.2025.104481), supporting the rationale for using two studies in the multivariable analysis.

The prognostic factors identified in the multivariable analysis of the stage III population, which included the MOSAIC and C-07 studies, were T and N stages, primary tumor side, age, number of examined lymph nodes, and presence of perforation/obstruction (Supplementary Table S2, available at https://doi.org/10.1016/j.esmoop.2025.104481).

#### T1-2 patients

The subgroup of T1 and T2 patients included 613 patients (12.4%). Preliminary analysis showed that survival outcomes were similar in T1 and T2 stage III patients, hence the two populations were combined. The main patient characteristics of the T1-2 subgroup according to treatment allocation were balanced except the subset of patients with BMI ≥30 who were significantly more prevalent in the experimental arm compared with the control arm (20% versus 28%, *P* = 0.027) (Supplementary Table S3, available at https://doi.org/10.1016/j.esmoop.2025.104481). Surprisingly, N stage was not prognostic in the T1-2 population; only age was an independent prognostic factor (Supplementary Table S4, available at https://doi.org/10.1016/j.esmoop.2025.104481).

#### T3 patients

The subgroup of T3 patients was the largest with 3677 patients (74.4%). Patient characteristics remained well balanced according to treatment allocation, except for the relative proportion of right-sided tumors that were significantly more prevalent in the OX arm than in the non-OX arm (44.4% versus 38.4%, *P =* .005) and age <70 years which was significantly more common in the OX arm than in the non-OX arm (83.3% versus 80.6%, *P =* 0.040) (Supplementary Table S5, available at https://doi.org/10.1016/j.esmoop.2025.104481). Independent prognostic factors in addition to treatment were N stage, age, primary tumor side, number of examined lymph nodes, and perforation/obstruction (Supplementary Table S6, available at https://doi.org/10.1016/j.esmoop.2025.104481).

#### T4 patients

The subgroup with T4 tumors included 646 patients (13.1%). Patient characteristics remained well balanced according to treatment allocation (Supplementary Table S7, available at https://doi.org/10.1016/j.esmoop.2025.104481). The single significant prognostic factor was N stage (Supplementary Table S8, available at https://doi.org/10.1016/j.esmoop.2025.104481).

#### N1 patients

Some 3198 patients (64.7%) were in this subgroup. Patient characteristics remained well balanced according to treatment allocation, except in the prevalence of right-sided tumors which were more common in the patients randomized to the OX arms compared with the non-OX arms (41.5% versus 36.0%, *P* = 0.016) (Supplementary Table S9, available at https://doi.org/10.1016/j.esmoop.2025.104481). Prognostic factors were T stage, age, the number of examined lymph nodes, and the presence of perforation/obstruction (Supplementary Table S10, available at https://doi.org/10.1016/j.esmoop.2025.104481).

#### N2 patients

A total of 1740 patients (35.3%) had N2 tumors. Patient characteristics remained well balanced according to treatment allocation (Supplementary Table S11, available at https://doi.org/10.1016/j.esmoop.2025.104481). Prognostic factors were T stage, age, primary tumor side, and the number of examined lymph nodes (Supplementary Table S12, available at https://doi.org/10.1016/j.esmoop.2025.104481).

### Effect of oxaliplatin on survival according to T and N stages

**Figure 2:**
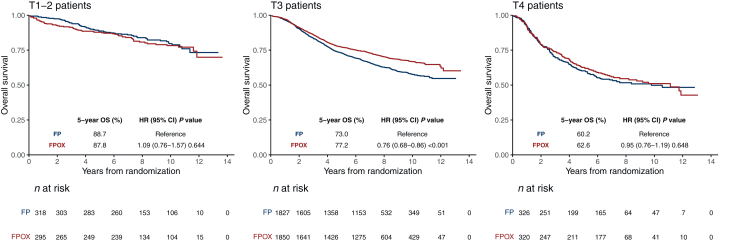
**Treatment group comparison regarding OS according to T stage.** CI, confidence interval; FP, fluoropyrimidine; FPOX, fluoropyrimidine and oxaliplatin; HR, hazard ratio; OS, overall survival.

**Figure 3:**
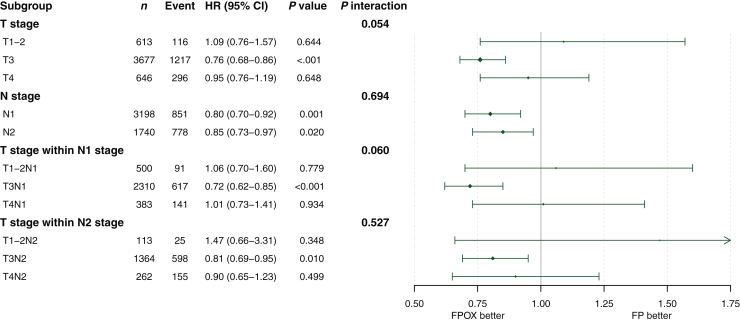
**Forest plot of the effect of OX use on OS in subgroups according to T and N stage in the pooled MOSAIC, C-07, and XELOXA studies.** CI, confidence interval; FP, fluoropyrimidine; FPOX, fluoropyrimidine and oxaliplatin; HR, hazard ratio; OS, overall survival; OX, oxaliplatin.

**Figure 4:**
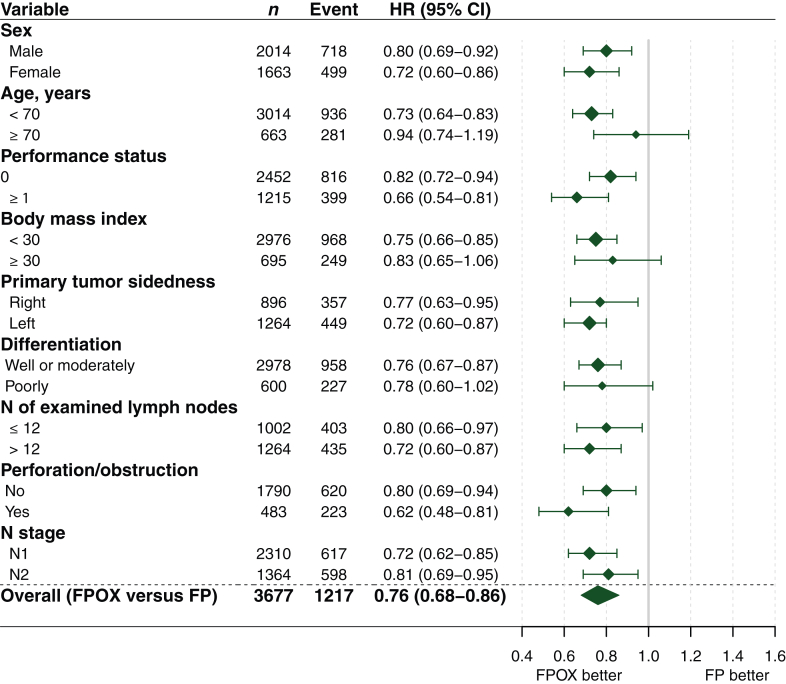
**Subgroup analysis of OS with OX in T3 stage colon cancer patients in the pooled MOSAIC, C-07, and XELOXA studies.** CI, confidence interval; FP, fluoropyrimidine; FPOX, fluoropyrimidine and oxaliplatin; HR, hazard ratio; OS, overall survival; OX, oxaliplatin.

An OS benefit of OX was observed in the T3 subgroup (HR 0.76, 95% CI 0.68-0.86, *P* < 0.001) but not in the T1-2 (HR 1.09, 95% CI 0.76-1.57, *P* = 0.644) or T4 (HR 0.95, 95% CI 0.76-1.19, *P* = 0.648) subgroups (Figure 2). Comparing FPOX with FP alone, the 5-year OS in T3 patients was 77.2% versus 73.0%, in T1-2 patients 87.8% versus 88.7%, and in T4 patients 62.6% versus 60.2% (Supplementary Table S13, available at https://doi.org/10.1016/j.esmoop.2025.104481). Interaction test between T stage and OX treatment effect on OS was significant (*P*_*int*_ = 0.054), whereas the interaction between N stage and OX treatment efficacy was not (*P*_*int*_ = 0.694), indicating that the effect of OX is more dependent on T stage than on N stage (Figure 3). The prevalence of the T3 subgroup (74.4%) could mask a differential effect of OX in the other subgroups. To confirm the varying treatment effects across T stages, we examined T stage within each of the N1 and N2 stages. In the N1 stage, a statistically significant OS benefit of OX was found in N1T3 patients, but not in N1T1-2 or N1T4. Similarly, in the N2 stage, a significant benefit of OX was observed in N2T3 patients, while no benefit was seen in N2T1-2 or N2T4 (Figure 3). Additionally, the TTR and DFS benefit of OX were noted in the T3 subpopulation, but not in T1-2 or T4 subpopulations. The interaction tests between T stage and N stage concerning OX treatment effects were not significant (Supplementary Figures S3 and S4, available at https://doi.org/10.1016/j.esmoop.2025.104481). OS Benefit of OX in the T3 subpopulation was consistent across most subgroups, including younger patients, those with good performance status, and left-sided tumors, but was less evident in patients aged ≥70 years or those with a BMI ≥30 (Figure 4).

### Validation of T stage as a superior prognostic factor

To further investigate the relative importance of T stage and N stage in our analysis, firstly, the results of a likelihood ratio test comparing nested models demonstrated that the inclusion of T stage significantly improved the model’s fit compared with the model that included only N stage (Supplementary Table S14, available at https://doi.org/10.1016/j.esmoop.2025.104481).

Secondly, our earlier analysis reported an HR of 0.82 for the overall patient cohort and 0.76 for T3 patients, both statistically significant. These findings can be utilized as a sensitivity analysis to support using OX in different tumor stages, with T3 patients showing a more pronounced benefit, highlighting T stage’s influence on treatment outcomes. Finally, significant interaction on OS has been observed between OX effect and stage II or III. Further interaction tests within the combined stage II and III populations revealed a significant OS interaction in the T3 subgroup but not in the T4 subgroup (Supplementary Table S15, available at https://doi.org/10.1016/j.esmoop.2025.104481). This suggests that the benefit of OX varies between stage II and stage III patients according to T stage, particularly showing in stage III an OS benefit in T3 patients but not in T4 patients. Specifically, in the adjuvant setting of the MOSAIC and C-07 studies, among T4 patients the addition of OX to FP regimens did not improve OS either in stage II disease (HR 0.96, 95% CI 0.56-1.66, *P* value = 0.890) or in stage III disease (HR 0.94, 95% CI 0.70-1.26, *P* value = 0.694).

### Efficacy of oxaliplatin in T4 subgroup

Regarding the outcomes for the T4 subgroup, the results concerning the potential benefit of OX in individual studies were divergent (Supplementary Table S16, available at https://doi.org/10.1016/j.esmoop.2025.104481). The discrepancy between a non-significant OS benefit in T4N2 substage reported in the MOSAIC (HR 0.75, 95% CI 0.45-1.26, *P* value = 0.275) and the lack of benefit in the two other studies (C-07, HR 0.91, 95% CI 0.44-1.85, *P* value = 0.787 and XELOXA, HR 1.05, 95% CI 0.64-1.70, *P* value = 0.854) prompted us to study the DFS and TTR in the T4N2 subgroup. The 5-year TTR and DFS were 59.6% and 58% in the T4N1 patients and 37.6% and 36.5% in the T4N2 patients, respectively. Non-significant 5-year TTR and DFS benefits of 8.3% and 7.2% for OX use in T4N1 and 8.0% and 8.5% in T4N2 were observed (Supplementary Figures S5 and S6, available at https://doi.org/10.1016/j.esmoop.2025.104481). The limited number of patients could explain here the lack of significance despite a meaningful absolute benefit.

The contrast between a possible benefit in TTR/DFS and the lack of benefit in OS in T4 could be attributed to differences in SAR (Supplementary Figure S7, available at https://doi.org/10.1016/j.esmoop.2025.104481). SAR depended on T stage, T1-3 versus T4 (HR_T1-2 versus T3_ = 0.85, 95% CI 0.68-1.07, *P* = 0.170 and HR_T4 versus T3_ = 1.35, 95% CI 1.18-1.54, *P* < 0.001). SAR also depended on the adjuvant treatment, with shorter post-relapse survival observed in the cohort that received FPOX versus FP alone. The observed difference in SAR was mostly driven by the T4 subgroup (Supplementary Figure S8, available at https://doi.org/10.1016/j.esmoop.2025.104481). This analysis has limitations, however, since the patients’ characteristics were different from the initial cohort and might not be balanced between the treatment groups.

## Discussion

T and N stage are the most potent independent prognostic factors in stage III CC.^17^ FPOX is currently the standardly prescribed adjuvant chemotherapy for stage III. This study evaluated survival outcomes with FP, with or without OX, across all stage III subgroups using individual patient data to conduct a pooled analysis of the three pivotal trials in making this comparison. OS was chosen for the main endpoint, but we also analyzed DFS that was the primary endpoint in the studies. In addition, TTR was also studied to avoid a bias due to death without recurrence, as 235 patients in our population (12%) had death without recurrence (a DFS event, but not a TTR event). In the MOSAIC trial, these patients have been reviewed, showing that the main causes of death without recurrence were second primary non-colorectal cancer and cardiovascular events.

Our findings underscored the nuanced role of OX in adjuvant treatment of stage III CC, particularly regarding tumor staging’s impact on outcomes. We observed a significant survival benefit for T3 patients, with an HR of 0.76, indicating a substantial reduction in mortality risk. No significant benefit was seen in T1-2 or T4 patients, however, suggesting that OX effectiveness may be limited to intermediate-stage tumors. The absence of OS benefit in T1-2 could be attributed to their favorable prognosis, with a 5-year OS of 88.3% consistent with a Surveillance, Epidemiology, and End Results (SEER) database analysis which reported a relative 5-year OS of 87.7% for patients with T1-2N1 and 75% for those with T1-2N2.^18^ OS benefit was not demonstrated in the T4 subgroup and the lack of interaction between stage II and stage III in this subgroup suggests the absence of an OX benefit on OS in all T4. In the T4 subgroup, the lack of significant OS benefits prompted further exploration of tumor recurrence and survival after relapse. While T4 patients may experience immediate benefits from OX, underlying factors could influence their survival, indicating a need for larger studies to validate these findings.

Our likelihood ratio test emphasized the importance of T stage in predicting outcomes, showing that its inclusion improves model fit compared with relying solely on N stage. This supports the need for tailored treatment strategies based on tumor characteristics. By highlighting the difference in HRs between the overall and T3-specific analyses, we underscored the critical role of tumor staging in optimizing therapeutic strategies for improved patient prognoses.

Following the IDEA study, which evaluated 3 versus 6 months of FOLFOX or CAPOX, 3 months of CAPOX was recommended for low-risk stage III patients, while 6 months of FOLFOX was advised for high-risk cases.^8^ While the IDEA classification helps to determine treatment duration, however, it does not adequately predict the benefit of OX, where T stage emerges as a critical factor. Notably, a study using the SEER database showed a greater weight of T stage over N stage in the staging of CC, the relative T and N stage weights being 0.58 and 0.42, respectively.^19^ The occurrence of circulating tumor cells also seems to better correlate with T stage than N stage, especially in left sided primary CC.^20^

In the two adjuvant trials evaluating irinotecan in the adjuvant setting that achieved negative results, authors reported that an imbalance in T4 favoring the control arm in both trials may have masked a significant advantage for the addition of irinotecan to LV5FU.^21^^,^^22^ The IROCAS (IRinotecan and Oxaliplatin for Colon Cancer in Adjuvant Setting) study is an ongoing trial in patients with high-risk stage III CC, comparing adjuvant modified FOLFIRINOX with modified-FOLFOX6 where patients are stratified according to T1-3N2, T4aN1, T4bN1, and T4N2. The results of this study, designed to define a role for the addition of irinotecan to modified FOLFOX6, could help to further assess the benefit of chemotherapy in the stage III subgroups.^23^ In our study, survival was poor in T4N2 patients, with a 5-year survival rate of 46.6%, which is close to the 51.2% 5-year survival rate in patients with resectable metastases.^24^

Among prognostic factors, perforation/obstruction, and tumor differentiation changed with T stage but not with N stage, tumor sidedness, age, and the number of examined lymph nodes. This and the differential effect of the addition of OX in the adjuvant chemotherapy of substage III CC suggest a difference in tumor biology between T stages. There might be a role of the tumor microenvironment with a decreased presence of lymphatic vessels and a reduced immune response that correlate with the increase in T stage.^25^^,^^26^

A recent paper addressed the issue of the relative benefit achieved with FP, OX, and chemotherapy duration for each subgroup of stage III CC defined by T and N.^27^ The goal of this study was to evaluate the individual contribution of each component of the adjuvant chemotherapy. The results questioned the benefit of OX when the expected benefit was small or clinically not relevant. The benefit of therapy, however, was based on the results of the individual adjuvant trials and a constant HR was considered for all T and N defined subgroups, while the benefit of OX is more likely to vary across subgroups. Here, we have shown that the prevalence of the T3N1-2 among stage III could mask a differential effect of OX in the other stage III subgroups.

In the T4N1-2 subgroup, the sample size (*n* = 645) might be too small to draw a definitive conclusion of lack of an OS benefit for OX. The HR in this group was 0.95 (*P* = 0.648). In the individual studies, the HRs of the T4 subgroups ranged between 0.75 and 1.20, and the benefit was variable among the studies. These patients could still have a meaningful benefit in TTR with an HR of 0.83 (*P* = 0.085) in the T4N1-2 patients. This non-significant benefit in TTR, however, did not translate into a benefit in OS. This finding correlated with a worse SAR in patients who received OX. Of note, the median TTR was shorter in the T4 cohort (13.9 months) than in the T3 (21.6 months) and T1-2 (25.8 months) cohorts. The probability of OX resistance is likely high when patients are retreated with OX after a relatively short TTR and this relative resistance may overshadow the TTR benefit.

The main strength of our pooled analysis is that it uses individual patient data derived from all stage III CC patients who were included in the three randomized large studies that compared adjuvant treatment with OX-containing regimens to those containing a single-agent FP treatment. There were detailed patient characteristics and outcome data available from these studies, all of which had long follow-up. Our study, however, had the limitations inherent in pooled analyses and there were a limited number of patients in some subgroups, decreasing the statistical power of those analyses, suggesting this work should be considered as hypothesis generating rather than definitive. There were no data in the pooled studies to permit the subdivision of patients with T4 tumors between T4a and T4b. In addition, in the XELOXA study there were no records of the primary tumor location, of tumor perforation/obstruction, or the exact number of positive lymph nodes. In addressing missing data for these variables, one potential approach is to introduce a ‘Missing’ category to retain these cases in the multivariable analysis. After further consideration, however, we determined that this method could introduce instability in the HR estimates. Stratifying Cox models by trial arms could exacerbate this issue, ultimately compromising the reliability of our findings. Therefore, we opted for a complete-case analysis to maintain the robustness and clarity of our results while acknowledging the limitations associated with missing data. The comparison of two multivariable models, MODEL 1 with a smaller set of widely available covariates and MODEL 2 with a larger set, despite missing data, demonstrated consistent results. This reinforced the rationale for pooling the MOSAIC and C-07 studies, as it enhances the interpretability and reliability of our conclusions regarding the impact of prognostic factors on patient outcomes.

With regard to the beneficial effects of OX in the adjuvant chemotherapy of stage III CC, the T stage appears to be more important than the N stage. Our results suggest that T stage could integrate the algorithm that clinicians apply when making the decision to add OX to a FP in individuals with stage III CC. The algorithm for adjuvant chemotherapy including our results remains 3 months of OX-based chemotherapy in T3N1, 3 or 6 months OX-based chemotherapy in T3N2, and individualized consideration for the use of an OX-based chemotherapy regimen in patients with T4N1 or T4N2 CC. For T1-2N1-2 cases, the benefit of adding OX to FP treatment should be questioned. In the near future, circulating tumor DNA may become a useful tool for estimating an individual patient’s prognosis and predicting the potential benefits of chemotherapy.^28^ Future studies using OX should prospectively stratify patients with stage III CC by T stage.
