# Supplementary material for: Impact of tumor and node stages on the efficacy of adjuvant oxaliplatin-based chemotherapy in stage III colon cancer patients: an ACCENT pooled analysis
Source: ESMO Open. 2025 Mar 4;10(3):104481. doi: 10.1016/j.esmoop.2025.104481 (PMC11928968; doi:10.1016/j.esmoop.2025.104481)

**Supplementary data**

Supplementary Table S1: Trials and treatment arms included in the analysis.

Supplementary Table S2: All stage III population, Overall survival univariable analysis (MOSAIC, C-07 and XELOXA) and multivariable analysis (MOSAIC and C-07)

Supplementary Table S3: Patient’s characteristics according to treatment in stage III T1-2 patients from the pooled MOSAIC, C-07 and XELOXA studies

Supplementary Table S4: Stage III T1-2 population, Overall survival univariable analysis (MOSAIC, C-07 - XELOXA) and multivariable analysis (MOSAIC and C-07)

Supplementary Table S5: Patient’s characteristics according to treatment in stage III T3 patients from the pooled MOSAIC, C-07 and XELOXA studies

Supplementary Table S6: Stage III T3 population, Overall survival univariable analysis (MOSAIC, C-07 and XELOXA) and multivariable analysis (MOSAIC and C-07)

Supplementary Table S7: Patient’s characteristics according to treatment in stage III T4 patients from the pooled MOSAIC, C-07 and XELOXA studies

Supplementary Table S8: Stage III T4 population, Overall survival univariable analysis (MOSAIC, C-07 and XELOXA) and multivariable analysis (MOSAIC and C-07)

Supplementary Table S9: Patient’s characteristics according to treatment in stage III N1 patients from the pooled MOSAIC, C-07 and XELOXA studies

Supplementary Table S10: Stage III N1 population, Overall survival univariable analysis (MOSAIC, C-07 and XELOXA) and multivariable analysis (MOSAIC and C-07)

Supplementary Table S11: Patient’s characteristics according to treatment in stage III N2 patients from the pooled MOSAIC, C-07 and XELOXA studies

Supplementary Table S12: Stage III N2 population, Overall survival univariable analysis (MOSAIC, C-07 and XELOXA) and multivariable analysis (MOSAIC and C-07)

Supplementary Table S13: The 5-year OS and 3-year TTR in patient subgroups defined with T stage.

Supplementary Table S14: Comparison of model fit using likelihood ratio test between T stage and N stage

Supplementary Table S15: Effect of the interaction between oxaliplatin use and stage disease (stage II and stage III) on OS in the pooled MOSAIC and C-07 studies.

Supplementary Table S16: The effect of OX use on OS in subgroups according to T4 and N stage in the MOSAIC, C-07 and XELOXA studies separately.

Supplementary Figure S1: T and N stages distribution in stage III according to treatment arm

Supplementary Figure S2: OS Comparison of Prognostic Factors in Stage III Population: Results from Multivariable Analysis

Supplementary Figure S3: Forest-plot of the effect of OX on TTR in subgroups according to T and N stage in the pooled MOSAIC, C-07 and XELOXA studies

Supplementary Figure S4: Forest-plot of the effect of OX on DFS in subgroups according to T and N stage in the pooled MOSAIC, C-07 and XELOXA studies

Supplementary Figure S5: Treatment group comparison regarding TTR in the T4N1-2 subgroup.

Supplementary Figure S6: Treatment group comparison regarding DFS in the T4N1-2 subgroup.

Supplementary Figure S7: Survival after relapse (SAR) in patient subgroup defined by T stage and oxaliplatin benefit on survival after relapse Stage III

Supplementary Figure S8: Oxaliplatin benefit on overall survival after relapse (SAR) in T1-2, T3 and T4 Stage III

This Appendix has been provided by the authors to provide readers additional information about their work.

**Supplementary Table S1:** **Trials and treatment arms included in the analysis (stage III disease).**

| **Study name** | **Years of accrual** | **Arm stratification text** | **Population study**  **N (%)** | **N** | | | | |
| --- | --- | --- | --- | --- | --- | --- | --- | --- |
|  |  |  |  | **N stage** | | **T stage** | | |
|  |  |  |  | **N1** | **N2** | **T1-2** | **T3** | **T4** |
| MOSAIC | 1998-2001 | FP | 675 (13.66) | 442 | 231 | 62 | 491 | 121 |
|  |  | FPOX | 671 (13.58) | 440 | 229 | 57 | 485 | 129 |
| C-07 | 2000-2002 | FP | 860 (17.40) | 554 | 306 | 157 | 638 | 61 |
|  |  | FPOX | 853 (17.26) | 543 | 310 | 133 | 665 | 54 |
| XELOXA | 2003-2004 | FP | 941 (19.04) | 608 | 333 | 99 | 698 | 144 |
|  |  | FPOX | 942 (19.06) | 611 | 331 | 105 | 700 | 137 |

N, number of patients; FP, fluoropyrimidine; FPOX, fluoropyrimidine and oxaliplatin.

**Supplementary Table S2: All stage III population, OS univariable analysis (MOSAIC, C-07 and XELOXA) and multivariable analysis (MOSAIC and C-07)**

|  |  | **Univariable analysis** | | | | **Multivariable analysis** | | |
| --- | --- | --- | --- | --- | --- | --- | --- | --- |
| **Variable** |  | ***N* (Event)** | **Hazard ratio** | **95%CI** | ***P*-value** | **Hazard ratio** | **95%CI** | ***P*-value** |
| **Gender** | Male | 2696 (937) | Reference |  |  |  |  |  |
|  | Female | 2246 (694) | 0.89 | [0.80-0.98] | **0.017** |  |  |  |
| **Age, y** | < 70 | 4051 (1255) | Reference |  |  | Reference |  |  |
|  | ≥ 70 | 891 (376) | 1.50 | [1.33-1.68] | **< 0.001** | 1.51 | [1.30-1.76] | < 0.001 |
| **ECOG PS** | 0 | 3241 (1055) | Reference |  |  |  |  |  |
|  | 1 | 1688 (573) | 1.10 | [0.98-1.24] | 0.11 |  |  |  |
| **BMI** | < 30 | 3990 (1318) | Reference |  |  |  |  |  |
|  | ≥ 30 | 944 (312) | 0.97 | [0.86-1.10] | 0.666 |  |  |  |
| **Sidedness** | Right | 1175 (460) | Reference |  |  | Reference |  |  |
|  | Left | 1715 (578) | 0.80 | [0.71-0.91] | **< 0.001** | 0.78 | [0.69-0.89] | < 0.001 |
| **Differentiation** | Other | 3930 (1242) | Reference |  |  |  |  |  |
|  | Poorly | 853 (335) | 1.40 | [1.24-1.58] | **< 0.001** |  |  |  |
| **T stage** | T1-2 | 613 (116) | Reference |  |  | Reference |  |  |
|  | T3 | 3677 (1217) | 2.05 | [1.69-2.48] | **< 0.001** | 2.14 | [1.68-2.72] | < 0.001 |
|  | T4 | 464 (296) | 3.45 | [2.77-4.29] | **< 0.001** | 3.12 | [2.34-4.17] | < 0.001 |
| **N stage** | N1 | 3198 (851) | Reference |  |  | Reference |  |  |
|  | N2 | 1740 (778) | 2.04 | [1.85-2.25] | **< 0.001** | 2.00 | [1.76-2.26] | < 0.001 |
| **N° of examined nodes** | > 12 | 1624 (559) | Reference |  |  | Reference |  |  |
|  | ≤ 12 | 1417 (536) | 1.12 | [0.99-1.26] | **0.067** | 1.39 | [1.23-1.58] | < 0.001 |
| **Perforation or Obstruction** | No | 2412 (804) | Reference |  |  | Reference |  |  |
|  | Yes | 639 (297) | 1.59 | [1.39-1.82] | **< 0.001** | 1.43 | [1.24-1.65] | < 0.001 |
| **Treatment** | FP | 2476 (884) | Reference |  |  | Reference |  |  |
|  | FPOX | 2466 (747) | 0.82 | [0.75-0.91] | **< 0.001** | 0.80 | [0.70-0.90] | < 0.001 |

**Note:** *P*-values are bolded for significant factors at the 0.1 level in the univariable analysis, which were included in the multivariable analysis.

Supplementary Table S3: Patient’s characteristics according to treatment in stage III T1-2 patients from the pooled MOSAIC, C-07 and XELOXA studies

|  | **Without oxaliplatin  (N=318)** | **With oxaliplatin  (N=295)** | **Stage III T1-2 (N=613)** | ***P*-value** |
| --- | --- | --- | --- | --- |
| **Age** |  |  |  | 0.46 |
| < 70 | 268 (84.28) | 241 (81.69) | 509 (83.03) |  |
| ≥ 70 | 50 (15.72) | 54 (18.31) | 104 (16.97) |  |
| **Gender** |  |  |  | 0.62 |
| Women | 137 (43.08) | 134 (45.42) | 271 (44.21) |  |
| Men | 181 (56.92) | 161 (54.58) | 342 (55.79) |  |
| **ECOG PS** |  |  |  | 0.22 |
| 0 | 233 (73.50) | 202 (68.71) | 435 (71.19) |  |
| 1+ | 84 (26.50) | 92 (31.29) | 176 (28.81) |  |
| Missing | 1 | 1 | 2 |  |
| **BMI** |  |  |  | **0.027** |
| < 30 | 229 (72.01) | 236 (80.00) | 465 (75.86) |  |
| ≥ 30 | 89 (27.99) | 59 (20.00) | 148 (24.14) |  |
| **Sidedness** |  |  |  | 0.68 |
| Left | 144 (68.25) | 123 (65.78) | 267 (67.09) |  |
| Right | 67 (31.75) | 64 (34.22) | 131 (32.91) |  |
| Missing | 107 | 108 | 215 |  |
| **Differentiation** |  |  |  | 0.35 |
| Poorly | 35 (11.51) | 41 (14.44) | 76 (12.93) |  |
| Well or Moderately | 269 (88.49) | 243 (85.56) | 512 (87.07) |  |
| Missing | 14 | 11 | 25 |  |
| **T stage** |  |  |  | 0.64 |
| T1 | 74 (23.27) | 63 (21.36) | 137 (23.35) |  |
| T2 | 244 (76.73) | 232 (78.64) | 476 (77.65) |  |
| **N stage** |  |  |  | 1 |
| N1 | 259 (81.45) | 241 (81.89) | 500 (81.57) |  |
| N2 | 59 (18.55) | 54 (18.31) | 113 (18.43) |  |
| **Perforation/Obstruction** |  |  |  | 0.49 |
| No | 204 (93.15) | 180 (95.24) | 384 (94.12) |  |
| Yes | 15 (6.85) | 9 (4.76) | 24 (5.88) |  |
| Missing | 99 | 106 | 205 |  |
| **Examined lymph nodes** |  |  |  | 0.12 |
| >12 | 78 (35.78) | 83 (43.92) | 161 (39.56) |  |
| ≤12 | 140 (64.22) | 106 (56.08) | 246 (60.44) |  |
| Missing | 100 | 106 | 206 |  |

**Supplementary Table S4:** **Stage III T1-2 population, OS univariable analysis (MOSAIC, C-07 and XELOXA) and multivariable analysis (MOSAIC and C-07)**

|  |  | **Univariable analysis** | | | | **Multivariable analysis** | | |
| --- | --- | --- | --- | --- | --- | --- | --- | --- |
| **Variable** |  | ***N* (Event)** | **Hazard ratio** | **95%CI** | ***P*-value** | **Hazard ratio** | **95%CI** | ***P*-value** |
| **Gender** | Male | 342 (66) | Reference |  |  |  |  |  |
|  | Female | 271 (50) | 0.97 | [0.67-1.41] | 0.891 |  |  |  |
| **Age, y** | < 70 | 509 (85) | Reference |  |  | Reference |  |  |
|  | ≥ 70 | 104 (31) | 1.93 | [1.28-2.92] | **0.002** | 1.90 | [1.13-3.19] | 0.015 |
| **ECOG PS** | 0 | 435 (78) | Reference |  |  |  |  |  |
|  | 1 | 176 (37) | 1.42 | [0.92-2.20] | 0.116 |  |  |  |
| **BMI** | < 30 | 465 (95) | Reference |  |  |  |  |  |
|  | ≥ 30 | 148 (21) | 0.66 | [0.41-1.06] | 0.082 |  |  |  |
| **Sidedness** | Right | 131 (30) | Reference |  |  |  |  |  |
|  | Left | 367 (46) | 0.74 | [0.46-1.18] | 0.203 |  |  |  |
| **Differentiation** | Other | 512 (100) | Reference |  |  |  |  |  |
|  | Poorly | 76 (11) | 0.72 | [0.39-1.35] | 0.309 |  |  |  |
| **N stage** | N1 | 500 (91) | Reference |  |  |  |  |  |
|  | N2 | 113 (25) | 1.28 | [0.82-2.00] | 0.268 |  |  |  |
| **N° of examined nodes** | > 12 | 161 (35) | Reference |  |  |  |  |  |
|  | ≤ 12 | 246 (42) | 0.75 | [0.48-1.18] | 0.214 |  |  |  |
| **Perforation or Obstruction** | No | 384 (76) | Reference |  |  |  |  |  |
|  | Yes | 24 (1) | 0.20 | [0.03-1.42] | 0.107 |  |  |  |
| **Treatment** | FP | 318 (58) | Reference |  |  |  |  |  |
|  | FPOX | 295 (54) | 1.09 | [0.76-1.57] | 0.644 |  |  |  |

**Note:** *P*-values are bolded for significant factors at the 0.1 level in the univariable analysis, which were included in the multivariable analysis.

Supplementary Table S5: Patient’s characteristics according to treatment in stage III T3 patients from the pooled MOSAIC, C-07 and XELOXA studies

|  | **Without oxaliplatin  (N=1827)** | **With oxaliplatin  (N=1850)** | **Stage III T3 (N=3677)** | ***P*-value** |
| --- | --- | --- | --- | --- |
| **Age** |  |  |  | **0.04** |
| < 70 | 1473 (80.62) | 1541 (83.30) | 3014 (81.97) |  |
| ≥ 70 | 354 (19.38) | 309 (16.70) | 663 (18.03) |  |
| **Gender** |  |  |  | 0.38 |
| Women | 840 (45.97) | 823 (44.49) | 1663 (45.23) |  |
| Men | 987 (54.03) | 1027 (55.51) | 2014 (54.77) |  |
| **ECOG PS** |  |  |  | 0.62 |
| 0 | 1212 (66.45) | 1240 (67.32) | 2452 (66.87) |  |
| 1+ | 612 (33.55) | 603 (32.68) | 1215 (33.13) |  |
| Missing | 3 | 7 | 10 |  |
| **BMI** |  |  |  | 0.70 |
| < 30 | 1483 (81.35) | 1493 (80.79) | 2976 (81.07) |  |
| ≥ 30 | 340 (18.65) | 355 (19.21) | 695 (18.93) |  |
| Missing | 4 | 2 | 6 |  |
| **Sidedness** |  |  |  | **0.005** |
| Left | 658 (61.61) | 606 (55.55) | 1264 (58.52) |  |
| Right | 411 (38.39) | 485 (44.45) | 896 (41.48) |  |
| Missing | 758 | 759 | 1516 |  |
| **Differentiation** |  |  |  | 0.10 |
| Poorly | 316 (17.82) | 284 (15.73) | 600 (16.77) |  |
| Well or Moderately | 1457 (82.18) | 1521 (84.27) | 2978 (83.23) |  |
| Missing | 54 | 45 | 99 |  |
| **N stage** |  |  |  | 1.00 |
| N1 | 1148 (62.90) | 1162 (62.84) | 2310 (62.87) |  |
| N2 | 677 (37.10) | 687 (37.16) | 1364 (37.13) |  |
| Missing | 2 | 1 | 3 |  |
| **Perforation/Obstruction** |  |  |  | 0.93 |
| No | 887 (78.63) | 903 (78.86) | 1790 (78.75) |  |
| Yes | 241 (21.37) | 242 (21.14) | 483 (21.25) |  |
| Missing | 699 | 705 | 1404 |  |
| **Examined lymph nodes** |  |  |  | 0.42 |
| >12 | 617 (54.89) | 647 (56.62) | 1264 (55.78) |  |
| ≤12 | 507 (45.11) | 495 (43.35) | 1002 (44.22) |  |
| Missing | 703 | 708 | 1411 |  |

**Supplementary Table S6:** **Stage III T3 population, OS univariable analysis (MOSAIC, C-07 and XELOXA) and multivariable analysis (MOSAIC and C-07)**

|  |  | **Univariable analysis** | | | | **Multivariable analysis** | | |
| --- | --- | --- | --- | --- | --- | --- | --- | --- |
| **Variable** |  | ***N* (Event)** | **Hazard ratio** | **95%CI** | ***P*-value** | **Hazard ratio** | **95%CI** | ***P*-value** |
| **Gender** | Male | 2014 (718) | Reference |  |  |  |  |  |
|  | Female | 1663 (499) | 0.83 | [0.74-0.93] | **0.002** |  |  |  |
| **Age, y** | < 70 | 3014 (936) | Reference |  |  | Reference |  |  |
|  | ≥ 70 | 663 (281) | 1.51 | [1.32-1.73] | **< 0.001** | 1.56 | [1.31-1.85] | < 0.001 |
| **ECOG PS** | 0 | 2452 (816) | Reference |  |  |  |  |  |
|  | 1 | 1215 (399) | 1.06 | [0.92-1.21] | 0.423 |  |  |  |
| **BMI** | < 30 | 2976 (968) | Reference |  |  |  |  |  |
|  | ≥ 30 | 695 (249) | 1.07 | [0.93-1.23] | 0.328 |  |  |  |
| **Sidedness** | Right | 896 (357) | Reference |  |  | Reference |  |  |
|  | Left | 1264 (449) | 0.83 | [0.73-0.96] | **0.010** | 0.75 | [0.65-0.87] | < 0.001 |
| **Differentiation** | Other | 2978 (958) | Reference |  |  |  |  |  |
|  | Poorly | 600 (227) | 1.28 | [1.11-1.48] | **0.001** |  |  |  |
| **N stage** | N1 | 2310 (617) | Reference |  |  | Reference |  |  |
|  | N2 | 1364 (598) | 1.94 | [1.74-2.18] | **< 0.001** | 2.02 | [1.75-2.33] | < 0.001 |
| **N° of examined nodes** | > 12 | 1264 (435) | Reference |  |  | Reference |  |  |
|  | ≤ 12 | 1002 (403) | 1.23 | [1.08-1.41] | **0.002** | 1.39 | [1.20-1.60] | < 0.001 |
| **Perforation or Obstruction** | No | 1790 (620) | Reference |  |  | Reference |  |  |
|  | Yes | 483 (223) | 1.48 | [1.27-1.73] | **< 0.001** | 1.53 | [1.31-1.80] | < 0.001 |
| **Treatment** | FP | 1827 (673) | Reference |  |  | Reference |  |  |
|  | FPOX | 1850 (544) | 0.76 | [0.68-0.86] | **< 0.001** | 0.75 | [0.65-0.86] | < 0.001 |

**Note:** *P*-values are bolded for significant factors at the 0.1 level in the univariable analysis, which were included in the multivariable analysis.

Supplementary Table S7: Patient’s characteristics according to treatment in stage III T4 patients from the pooled MOSAIC, C-07 and XELOXA studies

|  | **Without oxaliplatin  (N=326)** | **With oxaliplatin  (N=320)** | **Stage III T4  (N=646)** | ***P*-value** |
| --- | --- | --- | --- | --- |
| **Age** |  |  |  | 0.31 |
| < 70 | 270 (82.82) | 254 (79.38) | 524 (81.11) |  |
| ≥ 70 | 56 (17.18) | 66 (20.62) | 122 (18.89) |  |
| **Gender** |  |  |  | 0.47 |
| Women | 161 (49.39) | 148 (46.25) | 309 (47.83) |  |
| Men | 165 (50.61) | 172 (53.75) | 337 (52.17) |  |
| **ECOG PS** |  |  |  | 0.13 |
| 0 | 186 (57.23) | 163 (50.94) | 349 (54.11) |  |
| 1+ | 139 (42.77) | 157 (49.06) | 296 (45.89) |  |
| Missing | 1 | 0 | 1 |  |
| **BMI** |  |  |  | 0.56 |
| < 30 | 271 (83.64) | 274 (85.62) | 545 (84.63) |  |
| ≥ 30 | 53 (16.36) | 46 (14.37) | 99 (15.37) |  |
| Missing | 2 | 0 | 2 |  |
| **Sidedness** |  |  |  | 1.00 |
| Left | 88 (54.32) | 90 (54.88) | 178 (54.60) |  |
| Right | 74 (45.68) | 74 (45.12) | 148 (45.40) |  |
| Missing | 164 | 156 | 320 |  |
| **Differentiation** |  |  |  | 0.83 |
| Poorly | 86 (28.20) | 90 (29.32) | 176 (28.76) |  |
| Well or Moderately | 219 (71.80) | 217 (70.68) | 436 (71.24) |  |
| Missing | 21 | 13 | 34 |  |
| **N stage** |  |  |  | 0.99 |
| N1 | 193 (59.20) | 190 (59.56) | 383 (59.38) |  |
| N2 | 133 (40.80) | 129 (40.44) | 262 (40.62) |  |
| Missing | 0 | 1 | 1 |  |
| **Perforation/Obstruction** |  |  |  | 0.77 |
| No | 114 (62.98) | 119 (65.03) | 233 (64.01) |  |
| Yes | 67 (37.02) | 64 (34.97) | 131 (35.99) |  |
| Missing | 145 | 137 | 282 |  |
| **Examined lymph nodes** |  |  |  | 0.84 |
| >12 | 97 (53.89) | 101 (55.49) | 198 (54.70) |  |
| ≤12 | 83 (46.11) | 81 (44.51) | 164 (45.30) |  |
| Missing | 146 | 138 | 284 |  |

**Supplementary Table** **S8: Stage III T4 population, OS univariable analysis (MOSAIC, C-07 and XELOXA) and multivariable analysis (MOSAIC and C-07)**

|  |  | **Univariable analysis** | | | | **Multivariable analysis** | | |
| --- | --- | --- | --- | --- | --- | --- | --- | --- |
| **Variable** |  | ***N* (Event)** | **Hazard ratio** | **95%CI** | ***P*-value** | **Hazard ratio** | **95%CI** | ***P*-value** |
| **Gender** | Male | 337 (151) | Reference |  |  |  |  |  |
|  | Female | 309 (145) | 1.02 | [0.81-1.28] | 0.849 |  |  |  |
| **Age, y** | < 70 | 524 (233) | Reference |  |  |  |  |  |
|  | ≥ 70 | 122 (63) | 1.21 | [0.92-1.60] | 0.181 |  |  |  |
| **ECOG PS** | 0 | 349 (159) | Reference |  |  |  |  |  |
|  | 1 | 296 (137) | 0.99 | [0.76-1.29] | 0.957 |  |  |  |
| **BMI** | < 30 | 545 (254) | Reference |  |  |  |  |  |
|  | ≥ 30 | 99 (41) | 0.87 | [0.63-1.22] | 0.428 |  |  |  |
| **Sidedness** | Right | 148 (73) | Reference |  |  |  |  |  |
|  | Left | 178 (81) | 0.91 | [0.66-1.25] | 0.570 |  |  |  |
| **Differentiation** | Other | 436 (182) | Reference |  |  |  |  |  |
|  | Poorly | 176 (97) | 1.64 | [1.28-2.11] | **< 0.001** |  |  |  |
| **N stage** | N1 | 383 (141) | Reference |  |  | Reference |  |  |
|  | N2 | 262 (155) | 2.27 | [1.80-2.86] | **< 0.001** | 2.35 | [1.75-3.15] | < 0.001 |
| **N° of examined nodes** | > 12 | 198 (89) | Reference |  |  |  |  |  |
|  | ≤ 12 | 164 (89) | 1.27 | [0.95-1.70] | 0.112 |  |  |  |
| **Perforation or Obstruction** | No | 233 (106) | Reference |  |  |  |  |  |
|  | Yes | 131 (73) | 1.36 | [1.01-1.84] | **0.042** |  |  |  |
| **Treatment** | FP | 326 (151) | Reference |  |  |  |  |  |
|  | FPOX | 320 (145) | 0.95 | [0.75-1.19] | 0.648 |  |  |  |

**Note:** *P*-values are bolded for significant factors at the 0.1 level in the univariable analysis, which were included in the multivariable analysis.

Supplementary Table S9: Patient’s characteristics according to treatment in stage III N1 patients from the pooled MOSAIC, C-07 and XELOXA studies

|  | **Without oxaliplatin  (N=1604)** | **With oxaliplatin  (N=1594)** | **Stage III N1 (N=2998)** | ***P*-value** |
| --- | --- | --- | --- | --- |
| **Age** |  |  |  | 0.31 |
| < 70 | 1298 (80.92) | 1313 (82.37) | 2611 (81.64) |  |
| ≥ 70 | 306 (19.08) | 281 (17.63) | 587 (18.36) |  |
| **Gender** |  |  |  | 0.062 |
| Women | 751 (46.82) | 693 (43.48) | 1444 (45.15) |  |
| Men | 853 (53.18) | 901 (56.52) | 1754 (54.85) |  |
| **ECOG PS** |  |  |  | 0.69 |
| 0 | 1053 (65.73) | 1032 (65.99) | 2085 (65.36) |  |
| 1+ | 549 (34.27) | 556 (35.01) | 1105 (34.64) |  |
| Missing | 2 | 6 | 8 |  |
| **BMI** |  |  |  | 0.07 |
| < 30 | 1270 (79.33) | 1305 (81.92) | 2575 (80.62) |  |
| ≥ 30 | 331 (20.67) | 288 (18.08) | 619 (19.38) |  |
| Missing | 3 | 1 | 4 |  |
| **Sidedness** |  |  |  | **0.016** |
| Left | 602 (64.04) | 553 (58.52) | 1155 (61.27) |  |
| Right | 338 (35.96) | 392 (41.48) | 730 (38.73) |  |
| Missing | 664 | 649 | 1313 |  |
| **Differentiation** |  |  |  | 0.74 |
| Poorly | 219 (14.14) | 212 (13.66) | 431 (13.90) |  |
| Well or Moderately | 1330 (85.86) | 1340 (86.34) | 2670 (86.10) |  |
| Missing | 55 | 42 | 97 |  |
| **T stage** |  |  |  | 0.69 |
| T1-2 | 259 (16.19) | 241 (15.13) | 500 (15.66) |  |
| T3 | 1148 (71.75) | 1162 (72.94) | 2310 (72.35) |  |
| T4 | 193 (12.06) | 190 (11.93) | 383 (11.99) |  |
| Missing | 4 | 1 | 5 |  |
| **Perforation/Obstruction** |  |  |  | 0.66 |
| No | 786 (79.09) | 783 (79.98) | 1569 (79.52) |  |
| Yes | 208 (20.93) | 196 (20.02) | 404 (20.48) |  |
| Missing | 610 | 615 | 1225 |  |
| **Examined lymph nodes** |  |  |  | 0.05 |
| >12 | 444 (44.67) | 481 (49.18) | 925 (46.91) |  |
| ≤12 | 550 (55.33) | 497 (50.82) | 1047 (53.09) |  |
| Missing | 610 | 616 | 1226 |  |

**Supplementary Table S10:** **Stage III N1 population, OS univariable analysis (MOSAIC, C-07 and XELOXA) and multivariable analysis (MOSAIC and C-07)**

|  |  | **Univariable analysis** | | | | **Multivariable analysis** | | |
| --- | --- | --- | --- | --- | --- | --- | --- | --- |
| **Variable** |  | ***N* (Event)** | **Hazard ratio** | **95%CI** | ***P*-value** | **Hazard ratio** | **95%CI** | ***P*-value** |
| **Gender** | Male | 1754 (488) | Reference |  |  |  |  |  |
|  | Female | 1444 (363) | 0.91 | [0.79-1.04] | 0.166 |  |  |  |
| **Age, y** | < 70 | 2611 (649) | Reference |  |  | Reference |  |  |
|  | ≥ 70 | 587 (202) | 1.49 | [1.27-1.74] | **< 0.001** | 1.54 | [1.27-1.87] | < 0.001 |
| **ECOG PS** | 0 | 2085 (542) | Reference |  |  |  |  |  |
|  | 1 | 1105 (307) | 1.11 | [0.94-1.31] | 0.202 |  |  |  |
| **BMI** | < 30 | 2575 (690) | Reference |  |  |  |  |  |
|  | ≥ 30 | 619 (161) | 0.94 | [0.79-1.12] | 0.516 |  |  |  |
| **Sidedness** | Right | 730 (229) | Reference |  |  |  |  |  |
|  | Left | 1155 (331) | 0.87 | [0.74-1.03] | 0.113 |  |  |  |
| **Differentiation** | Other | 2670 (711) | Reference |  |  |  |  |  |
|  | Poorly | 431 (116) | 1.03 | [0.84-1.25] | 0.777 |  |  |  |
| **T stage** | T1-2 | 500 (91) | Reference |  |  | Reference |  |  |
|  | T3 | 2310 (617) | 1.65 | [1.32-2.06] | **< 0.001** | 1.78 | [1.36-2.34] | < 0.001 |
|  | T4 | 383 (141) | 2.57 | [1.96-3.36] | **< 0.001** | 2.54 | [1.82-3.55] | < 0.001 |
| **N° of examined nodes** | > 12 | 925 (244) | Reference |  |  | Reference |  |  |
|  | ≤ 12 | 1047 (340) | 1.29 | [1.09-1.52] | **0.003** | 1.34 | [1.13-1.58] | 0.001 |
| **Perforation or Obstruction** | No | 1569 (420) | Reference |  |  | Reference |  |  |
|  | Yes | 404 (164) | 1.74 | [1.45-2.08] | **< 0.001** | 1.59 | [1.32-1.91] | < 0.001 |
| **Treatment** | FP | 1604 (467) | Reference |  |  | Reference |  |  |
|  | FPOX | 1594 (384) | 0.80 | [0.70-0.92] | **0.001** | 0.83 | [0.71-0.98] | 0.028 |

**Note:** *P*-values are bolded for significant factors at the 0.1 level in the univariable analysis, which were included in the multivariable analysis.

Supplementary Table S11: Patient’s characteristics according to treatment in stage III N2 patients from the pooled MOSAIC, C-07 and XELOXA studies

|  | **Without oxaliplatin  (N=870)** | **With oxaliplatin  (N=870)** | **Stage III N2 (N=1740)** | ***P*-value** |
| --- | --- | --- | --- | --- |
| **Age** |  |  |  | 0.66 |
| < 70 | 714 (82.07) | 722 (82.99) | 1436 (82.53) |  |
| ≥ 70 | 156 (17.93) | 148 (17.01) | 304 (17.47) |  |
| **Gender** |  |  |  | 0.29 |
| Women | 388 (44.60) | 411 (47.24) | 799 (45.92) |  |
| Men | 482 (55.40) | 459 (52.76) | 941 (54.08) |  |
| **ECOG PS** |  |  |  | 0.62 |
| 0 | 583 (67.24) | 573 (66.01) | 1156 (66.63) |  |
| 1+ | 284 (32.76) | 295 (33.99) | 579 (33.37) |  |
| Missing | 3 | 2 | 5 |  |
| **BMI** |  |  |  | 0.28 |
| < 30 | 714 (82.35) | 697 (80.21) | 1411 (81.28) |  |
| ≥ 30 | 153 (17.65) | 172 (19.79) | 325 (18.72) |  |
| Missing | 3 | 1 | 4 |  |
| **Sidedness** |  |  |  | 0.17 |
| Left | 293 (57.91) | 265 (53.43) | 558 (55.69) |  |
| Right | 213 (42.09) | 231 (46.57) | 444 (44.31) |  |
| Missing | 364 | 374 | 738 |  |
| **Differentiation** |  |  |  | 0.34 |
| Poorly | 219 (26.23) | 203 (24.08) | 422 (25.15) |  |
| Well or Moderately | 616 (73.77) | 640 (75.92) | 1256 (74.85) |  |
| Missing | 35 | 27 | 62 |  |
| **T stage** |  |  |  | 0.84 |
| T1-2 | 59 (6.79) | 54 (15.13) | 113 (6.50) |  |
| T3 | 677 (77.91) | 687 (78.97) | 1364 (78.44) |  |
| T4 | 133 (15.30) | 129 (14.83) | 262 (15.07) |  |
| Missing | 1 | 0 | 1 |  |
| **Perforation/Obstruction** |  |  |  | 0.71 |
| No | 423 (78.77) | 417 (77.65) | 840 (78.21) |  |
| Yes | 114 (21.23) | 120 (23.35) | 234 (21.79) |  |
| Missing | 333 | 333 | 666 |  |
| **Examined lymph nodes** |  |  |  | 1 |
| >12 | 349 (65.48) | 350 (65.30) | 699 (65.39) |  |
| ≤12 | 184 (34.52) | 186 (34.70) | 370 (34.61) |  |
| Missing | 337 | 334 | 671 |  |

**Supplementary Table S12:** **Stage III N2 population, univariable analysis (MOSAIC, C-07 and XELOXA) and multivariable analysis (MOSAIC and C-07)**

|  |  | **Univariable analysis** | | | | **Multivariable analysis** | | |
| --- | --- | --- | --- | --- | --- | --- | --- | --- |
| **Variable** |  | ***N* (Event)** | **Hazard ratio** | **95%CI** | ***P*-value** | **Hazard ratio** | **95%CI** | ***P*-value** |
| **Gender** | Male | 941 (448) | Reference |  |  |  |  |  |
|  | Female | 799 (330) | 0.85 | [0.73-0.98] | **0.022** |  |  |  |
| **Age, y** | < 70 | 1436 (604) | Reference |  |  | Reference |  |  |
|  | ≥ 70 | 304 (174) | 1.61 | [1.36-1.90] | **< 0.001** | 1.44 | [1.14-1.81] | 0.002 |
| **ECOG PS** | 0 | 1156 (513) | Reference |  |  |  |  |  |
|  | 1 | 579 (264) | 1.12 | [0.94-1.32] | 0.214 |  |  |  |
| **BMI** | < 30 | 1411 (626) | Reference |  |  |  |  |  |
|  | ≥ 30 | 325 (151) | 1.04 | [0.87-1.24] | 0.687 |  |  |  |
| **Sidedness** | Right | 444 (231) | Reference |  |  | Reference |  |  |
|  | Left | 558 (246) | 0.77 | [0.64-0.92] | **0.005** | 0.73 | [0.61-0.88] | 0.001 |
| **Differentiation** | Other | 1256 (529) | Reference |  |  |  |  |  |
|  | Poorly | 422 (219) | 1.49 | [1.27-1.75] | **< 0.001** |  |  |  |
| **T stage** | T1-2 | 113 (25) | Reference |  |  | Reference |  |  |
|  | T3 | 1364 (598) | 2.45 | [1.64-3.66] | **< 0.001** | 3.91 | [2.20-6.96] | < 0.001 |
|  | T4 | 262 (155) | 4.54 | [2.96-6.96] | **< 0.001** | 7.02 | [3.78-13.04] | < 0.001 |
| **N° of examined nodes** | > 12 | 699 (315) | Reference |  |  | Reference |  |  |
|  | ≤ 12 | 370 (196) | 1.25 | [1.05-1.49] | **0.014** | 1.39 | [1.15-1.67] | 0.001 |
| **Perforation or Obstruction** | No | 840 (382) | Reference |  |  |  |  |  |
|  | Yes | 234 (133) | 1.41 | [1.15-1.71] | **0.001** |  |  |  |
| **Treatment** | FP | 870 (416) | Reference |  |  | Reference |  |  |
|  | FPOX | 870 (362) | 0.85 | [0.75-0.97] | **0.02** | 0.78 | [0.65-0.94] | 0.008 |

**Note:** *P*-values are bolded for significant factors at the 0.1 level in the univariable analysis, which were included in the multivariable analysis.

**Supplementary Table S13: The 5-year OS and 3-year TTR in patient subgroups defined with T stage.**

|  |  |  | **OS** | | | | **TTR** | | | |
| --- | --- | --- | --- | --- | --- | --- | --- | --- | --- | --- |
|  |  |  | 5-year | | HR (95% CI) | *P*-value | 3-year | | HR (95% CI) | *P*-value |
| **Subgroup** | | *N* | FP | FPOX |  |  | FP | FPOX |  |  |
| **T1-2** | All | 613 | 88.7 | 87.8 | 1.09 (0.76-1.57) | .644 | 86.7 | 87.4 | 0.95 (0.65-1.38) | .783 |
|  | N1 | 500 | 89.7 | 89.1 | 1.06 (0.70-1.60) | .779 | 89.2 | 88.7 | 0.95 (0.62-1.44) | .802 |
|  | N2 | 113 | 84.2 | 82.0 | 1.47 (0.66-3.31) | .348 | 80.5 | 81.4 | 1.07 (0.47-2.47) | .868 |
| **T3** | All | 3677 | 73.0 | 77.2 | 0.76 (0.68-0.86) | <.001 | 68.2 | 73.7 | 0.75 (0.67-0.83) | <.001 |
|  | N1 | 2310 | 79.1 | 83.8 | 0.72 (0.62-0.85) | <.001 | 75.4 | 80.7 | 0.71 (0.60-0.82) | <.001 |
|  | N2 | 1364 | 62.5 | 66.1 | 0.81 (0.69-0.95) | .010 | 56.1 | 62.1 | 0.78 (0.67-0.92) | .002 |
| **T4** | All | 646 | 60.2 | 62.6 | 0.95 (0.76-1.19) | .648 | 52.4 | 62.2 | 0.83 (0.66-1.03) | .085 |
|  | N1 | 383 | 71.3 | 71.2 | 1.01 (0.73-1.41) | .934 | 60.0 | 72.1 | 0.80 (0.59-1.10) | .167 |
|  | N2 | 262 | 43.7 | 49.5 | 0.90 (0.65-1.23) | .499 | 41.3 | 47.3 | 0.84 (0.61-1.14) | .255 |

Abbreviations: HR, hazard ratio in FPOX vs FP; 95%CI, 95% confidence interval

**Supplementary Table S14: Comparison of model fit using likelihood ratio test between T stage and N stag**

| **Model** | **Log-Likelihood** | **Chi-Square** | **Degrees of Freedom** | ***P*-value** |
| --- | --- | --- | --- | --- |
| Model including T stage | -11.502 |  |  |  |
| Model including N stage | -11.471 | 60.072 | 1 | <.001 |
| Test | ANOVA |  |  |  |

**Supplementary Table S15: Effect of the interaction between oxaliplatin use and stage disease (stage II and stage III) on OS in the pooled MOSAIC and C-07 studies.**

|  | **N, HR (95% CI) *P*-value** | |  | ***P*-value of interaction** |
| --- | --- | --- | --- | --- |
|  | **Stage II** | **Stage III** |  |  |
| **All patients** | 1596, 1.03 (0.82-1.29) .813 | 4323, 0.82 (0.73-0.92) .001 |  | .073 |
| **T3** | 1375, 1.04 (0.82-1.33) .740 | 3677, 0.76 (0.66-0.87) <.001 |  | .022 |
| **T4** | 221, 0.96 (0.56-1.66) .890 | 646, 0.94 (0.70-1.26) .694 |  | .954 |

**Supplementary Table S16: The effect of OX use on OS in subgroups according to T4 and N stage in the MOSAIC, C-07 and XELOXA studies separately.**

|  | **MOSAIC** | | **C-07** | | **XELOXA** | |
| --- | --- | --- | --- | --- | --- | --- |
| **Subgroup** | **N HR (95%CI)** | ***P-value*** | **N HR (95%CI)** | ***P-value*** | **N HR (95%CI)** | ***P-value*** |
| T4 | 250 0.90 (0.63-1.29) | 0.573 | 115 1.03 (0.62-1.70) | 0.911 | 281 0.96 (0.66-1.38) | 0.811 |
| T4N1 | 153 1.16 (0.70-1.94) | 0.565 | 721.20 (0.58-2.45) | 0.626 | 158 0.78 (0.45-1.37) | 0.39 |
| T4N2 | 96 0.75 (0.45-1.26) | 0.275 | 43 0.91 (0.44-1.85) | 0.787 | 123 1.05 (0.64-1.70) | 0.854 |

Abbreviations: N, number of patient; HR, hazard ration FPOX vs FP; 95%CI, 95% confidence interval

**Supplementary Figure S1:** **T and N stages distribution in stage III according to treatment arm**


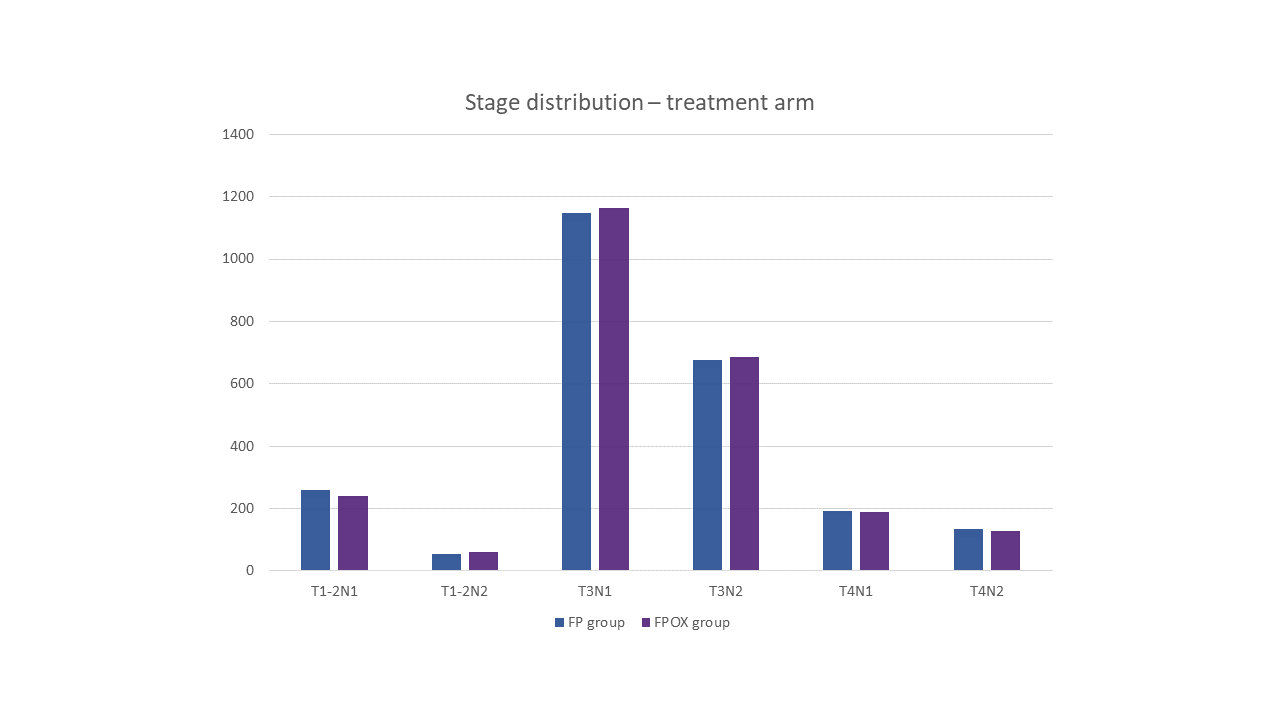


**Supplementary Figure S2: OS Comparison of Prognostic Factors in Stage III Population: Results from Multivariable Analysis**


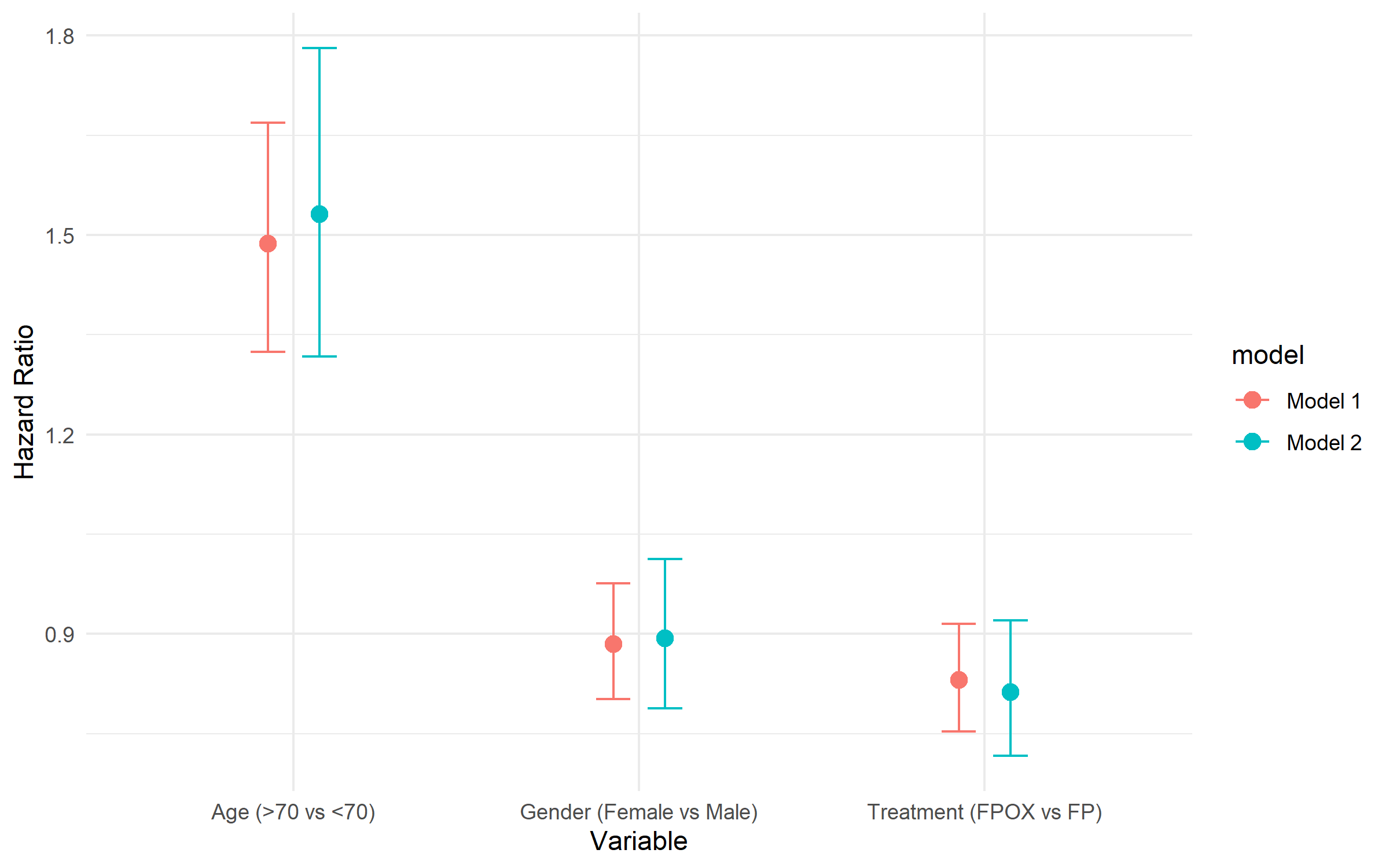


Model 1: Smaller set of adjusting variables (widely available across trials)

Model 2: Larger set of adjusting variables (including additional predictors, limited by missing data)

**Supplementary Figure S3: Forest-plot of the effect of OX on TTR in subgroups according to T and N stage in the pooled MOSAIC, C-07 and XELOXA studies**


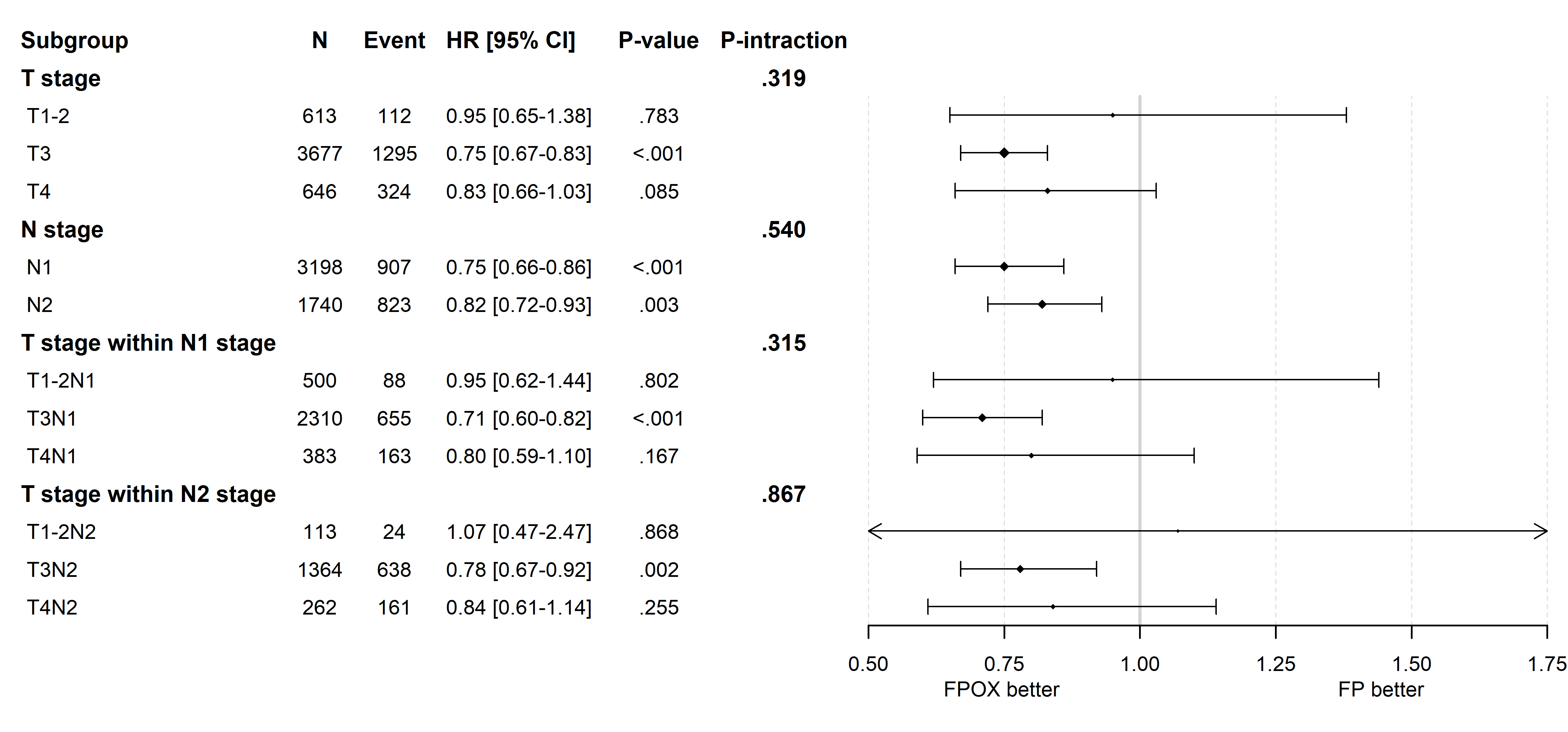


**Supplementary Figure S4: Forest-plot of the effect of OX on DFS in subgroups according to T and N stage in the pooled MOSAIC, C-07 and XELOXA studies**


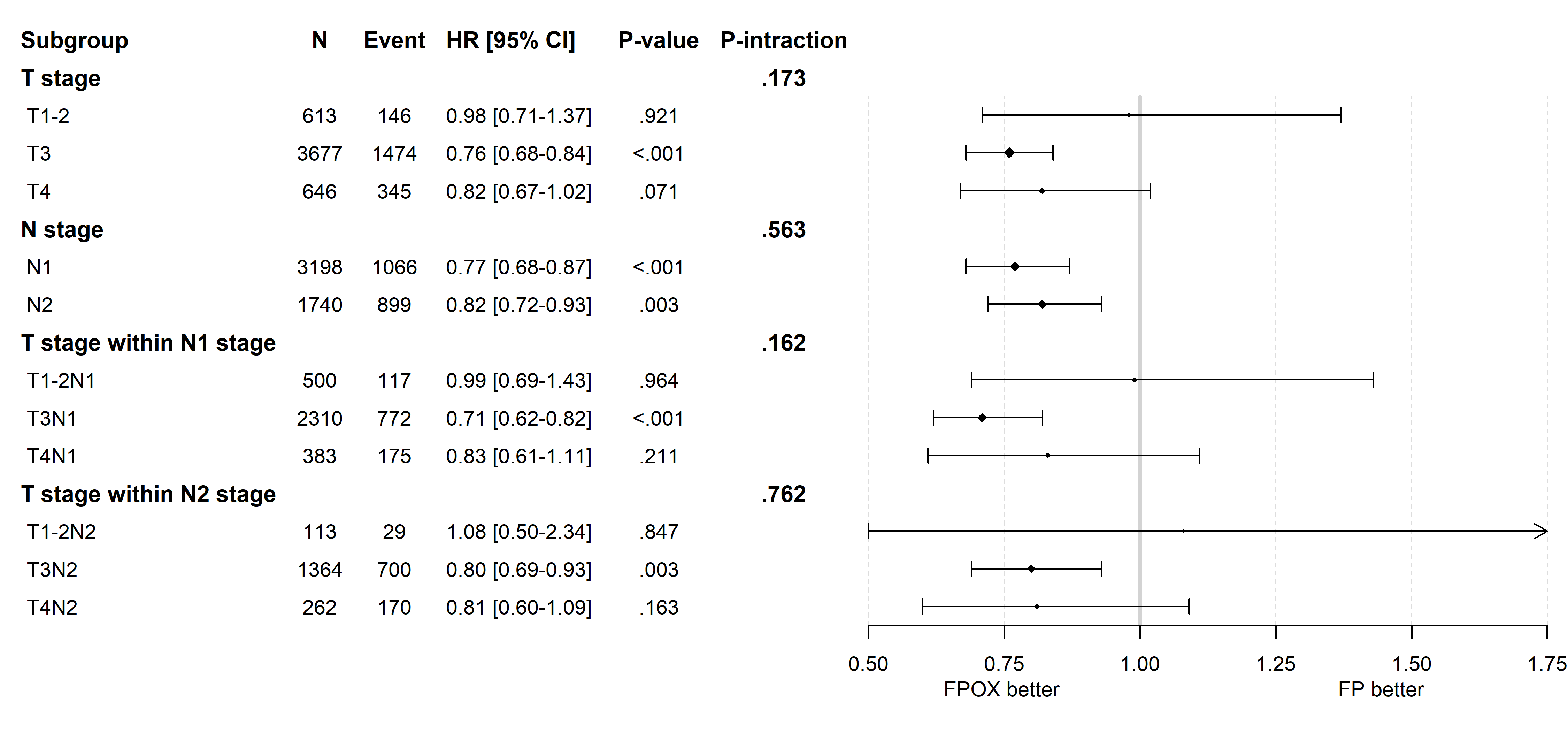


**Supplementary Figure S5: Treatment group comparison regarding TTR in the T4N1-2 subgroup.**

**
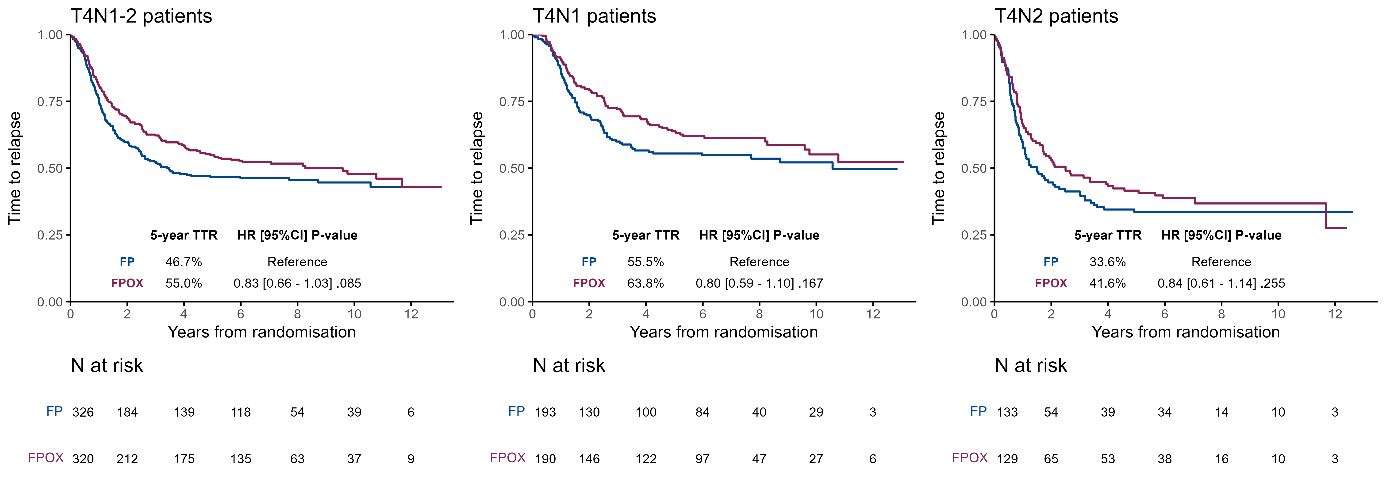
**

**Supplementary Figure S6: Treatment group comparison regarding DFS in the T4N1-2 subgroup.**


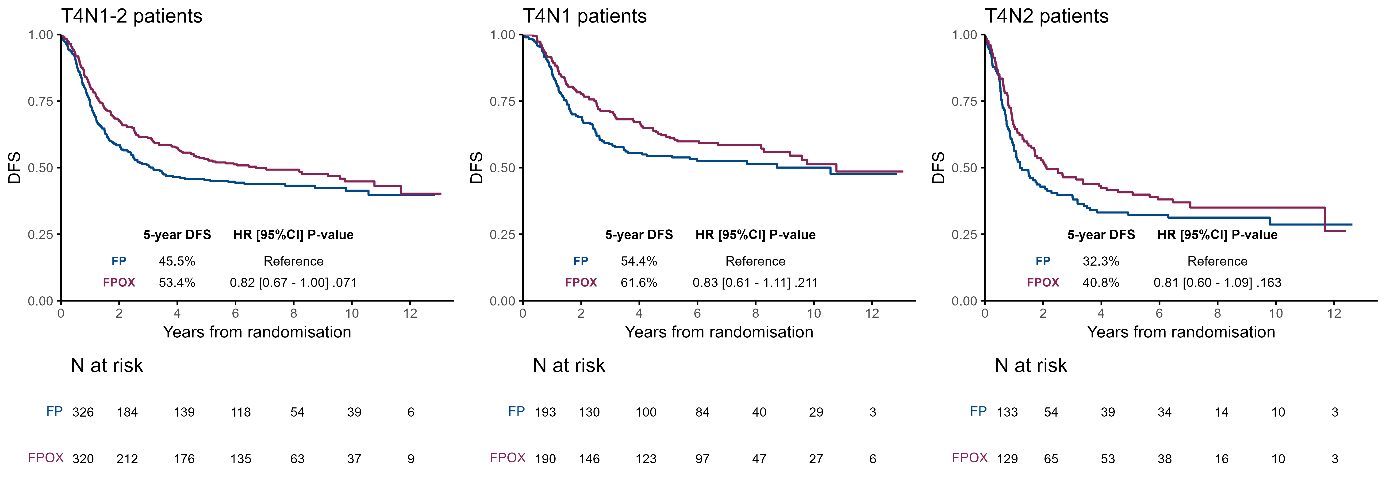


**Supplementary Figure S7: Survival after relapse (SAR) in patient subgroup defined by T stage and oxaliplatin benefit on SAR Stage III**


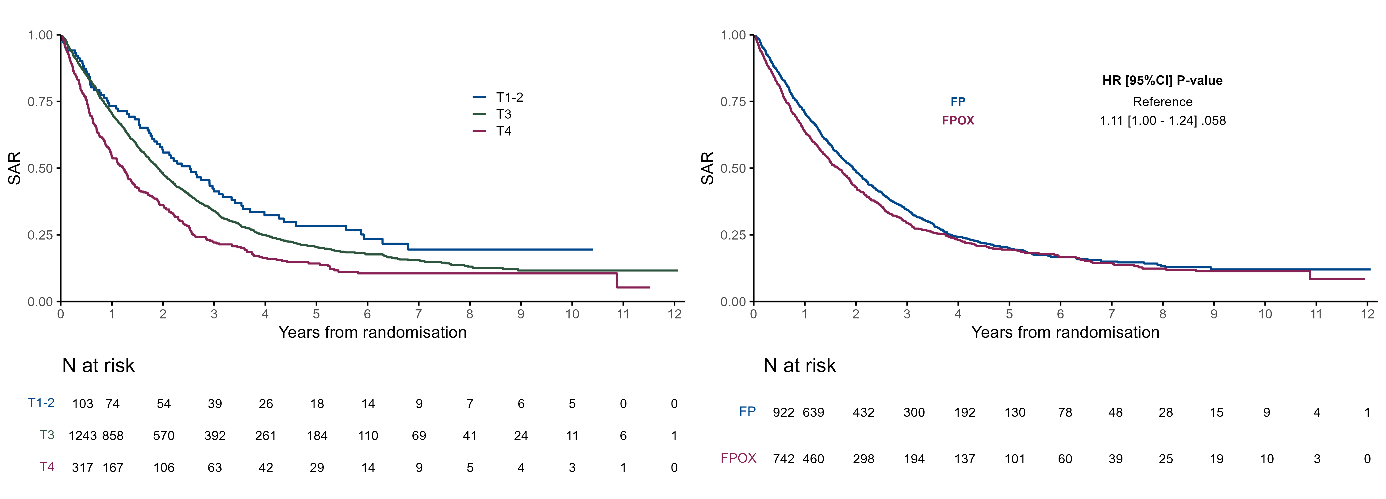


**Supplementary Figure S8: Oxaliplatin benefit on survival after relapse (SAR) in T1-2, T3 and T4 Stage III**


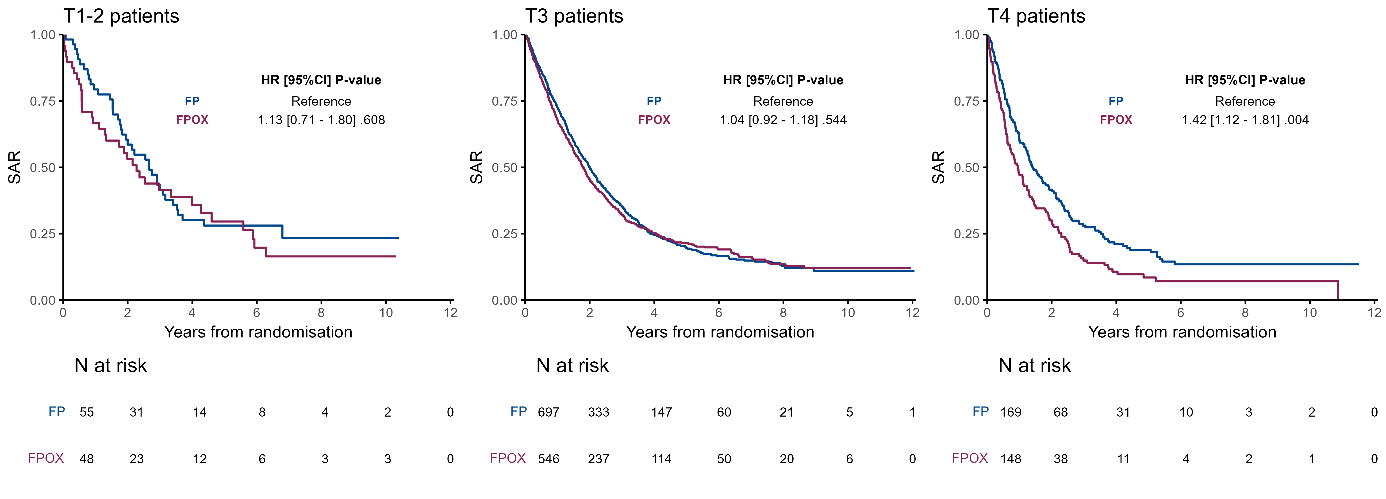

Supplement: Supplementary Appendix [file mmc1.docx]
